# Costs of injury for scent signalling in a strepsirrhine primate

**DOI:** 10.1038/s41598-018-27322-3

**Published:** 2018-06-29

**Authors:** Rachel L. Harris, Marylène Boulet, Kathleen E. Grogan, Christine M. Drea

**Affiliations:** 1 Department of Evolutionary Anthropology, Duke University, Durham, NC USA; 2 Department of Biology, Bishop’s University, Sherbrooke, QC Canada; 3 Department of Anthropology, Pennsylvania State University, State College, PA USA; 4 Department of Biology, Duke University, Durham, NC USA

## Abstract

Honesty is crucial in animal communication when signallers are conveying information about their condition. Condition dependence implies a cost to signal production; yet, evidence of such cost is scarce. We examined the effects of naturally occurring injury on the quality and salience of olfactory signals in ring-tailed lemurs (*Lemur catta*). Over a decade, we collected genital secretions from 23 (13 male, 10 female) adults across 34 unique injuries, owing primarily to intra-group fights. Using gas chromatography-mass spectrometry, we tested for differences in the chemical composition of secretions across pre-injury, injury and recovery, in animals that did and did not receive antibiotics. Lemur genital secretions were significantly dampened and altered during injury, with patterns of change varying by sex, season and antibiotics. Using behavioural bioassays (excluding odorants from antibiotic-treated animals), we showed that male ‘recipients’ discriminated injury status based on scent alone, directing more competitive counter marking towards odorants from injured vs. uninjured male ‘signallers.’ That injured animals could not maintain their normal signatures provides rare evidence of the energetic cost to signal production. That conspecifics detected olfactory-encoded ‘weakness’ suggests added behavioural costs: By influencing the likelihood of intra- or inter-sexual conflict, condition-dependent signals could have important implications for socio-reproductive behaviour.

## Introduction

For animal communication to effectively guide social behaviour, competition and mate choice, the signals used require a degree of honesty or ‘condition-dependence’^1,2^. Examples of condition dependency in signals and cues, including in vocalisations, visual ornaments, weaponry, behavioural displays and odours, are found throughout the animal kingdom^1,3^. Moreover, signal receivers use variations in signal composition and quality to assess both the stable and transient condition of conspecific signallers, altering their behavioural responses accordingly^3–5^. Because olfactory signals or cues are inextricably tied to an animal’s underlying physiology (perhaps even more so than are signals in other modalities), they are thought to provide a particularly reliable avenue for the advertisement and assessment of transient health, body condition or infection status^6–9^. Although evidence of condition dependence may imply a cost to scent production^6,10^, definitive evidence of such a cost (independent of pathogenic infection) is difficult to obtain without directly manipulating the physical condition of the signaller. Moreover, researchers rarely incorporate both chemical and behavioural methods in the same study. Using a strepsirrhine primate, the ring-tailed lemur (*Lemur catta*), we test for salient, condition-dependent variation in genital odorants associated with periods of wellness versus periods of naturally occurring injury. Specifically, we combined gas chromatography-mass spectrometry (GC-MS) with behavioural testing to examine (1) if lemur odorants vary chemically with injury and (2) if conspecifics are sensitive to such changes.

Indicator models of sexual selection predict that the expression of exaggerated signals should be condition-dependent, honestly conveying information about the signalling animal^11–13^. The expression of condition-dependent traits positively correlates with an individual’s acquired pool of resources^13^ and ability to withstand environmental challenges^12^, thus reflecting the degree to which an individual is impacted by poor nutrition, parasite load or physiological stress^3,14^. The maintenance of honesty in condition-dependent signals is a topic of current debate^2,15,16^ centring around two overarching, non-mutually exclusive principles: the ‘costly signaling hypothesis’ and the ‘index hypothesis’ (summarised by Weaver^16^).

Developed from Zahavi’s handicap principle^17^, the ‘costly signalling hypothesis’ posits that (1) signals are kept honest by production costs paid by the signaller and (2) low-condition individuals experience relatively greater costs than do individuals in good condition^11,18^. Recent critics^2,15^ argue that a handicap is not necessary to maintain signal honesty. Instead, signals may be kept honest, not by realised costs paid by honest signallers, but by the potential costs differentially paid by cheaters. For example, if the cost to signal production (however small) outweighs any benefit from investing in that cost, then ‘low-quality’ individuals will not likely cheat to produce a dishonest, ‘high-quality’ signal^6,19^. Researchers have variously expanded the definition of costly signalling to incorporate trade-offs associated with immunocompetence^20^, resource allocation^13^ and oxidative stress^4^.

In contrast to the handicap hypothesis, the ‘index hypothesis’^2^ does not require that honest signals be costly to produce. Instead, honesty is maintained by condition-dependent signals being mechanistically tied to a genetic or physiological pathway that is impossible to circumvent^12,14,21^. Regardless of the specific mechanism, evidence of condition-dependent, sexually selected traits derives overwhelmingly from studies of male visual^5,16^ and vocal^22^ signals. Nevertheless, the same principles may be applied to less easily measured traits, such as behavioural displays or olfactory signals^23^, including in females.

To communicate and coordinate sociality and reproduction, many vertebrates rely on complex chemical blends released from excretory products, saliva and scent glands^7,24^. Condition-dependent odorants and associated scent-marking behaviour are often sexually selected^24^, honestly conveying information on signaller traits, including sex, identity, age, reproductive state, dominance status and genetic quality^7,24^. Owing to their intimate ties to internal physiology, odorants are thought to be particularly sensitive to fluctuations in signaller health^6,7^. Thus, in addition to the life-history costs associated with signalling effort, experienced by any scent-marking species^25^, examples of condition-dependent odorant production and scent-marking activity have been found in lizards^8,26^, herpestids^27^ and laboratory rodents^28–30^. In the latter, researchers have shown that male mice inoculated with a pathogen or virus tend to invest less in scent-marking behaviour and produce depleted olfactory signals, and that female conspecifics prefer odorants from healthy males over those from infected males. Similar results are even obtained by challenging the immune system with non-replicating bacteria or lipopolysaccharides^10,31,32^. Such depleted investment in olfactory signals by immune-challenged animals implies an energetic cost to odorant production, creating a trade-off in investment between survival, reproduction and ornamentation^10^. To further our understanding of the condition dependence and potential costs of animal signals, it is crucial to consider other types of condition (beyond infection) that might affect energetic resources available for signal production, such as poor nutrition or injury.

Acute injury induces immediate, physiological responses from the mammalian immune and neuroendocrine systems^33,34^, both of which are known to affect the expression of sexually selected traits^35^. Nevertheless, experimental evidence of olfactory communication being influenced by injury is limited to the behavioural responses of bystanders to ‘alarm pheromones,’ which function as cues of predator-induced injury in conspecifics (e.g., flatworms^36^, mollusks^37^, crustaceans^38^, insects^39^ and fish^40^). Recently, Kimball and colleagues^41^ also showed that experimentally injured mice produce altered urinary cues that are salient to conspecifics; however, the authors were focused on developing an olfactory diagnostic tool for human brain injury and not on the implications of injury for rodent social communication. Despite the potentially serious physical and socio-reproductive consequences of injury (e.g., decreased competitive ability or social status, loss of access to potential mates), its effects on condition-dependent olfactory signals and on the behaviour of signal recipients remain to be described in natural systems.

In this study, we examined the effects of natural injuries on scent signatures and conspecific responses to such odorants in a socially complex mammal. Living in female-dominant, multi-male multi-female groups^42^, the ring-tailed lemur is an ideal model in which to examine the condition dependence of olfactory signals in both sexes. Ring-tailed lemurs arguably possess the most elaborate olfactory repertoire of any primate^42–44^: Both sexes possess scent glands (that are unique in the male), engage in conspicuous, multimodal scent-marking behaviour, and deposit chemically elaborate bouquets that contain information on the signaller’s sex, reproductive condition (e.g., breeding season, hormonal state), individual identity, neutral heterozygosity, diversity at the Major Histocompatibility Complex (MHC) and kinship^43,45–49^. Moreover, as a strepsirrhine with a functional vomeronasal organ^50^ and a derived increase in olfactory sensitivity^51^, ring-tailed lemurs of both sexes discriminate between different types of conspecific glandular secretions, modulating their behavioural responses seasonally and depending upon characteristics of the signaller^45,48,49,52,53^. Lastly, genital secretions in both sexes show salient, season-specific and potentially stress-induced decrements in chemical diversity (i.e., reflecting transient condition), that are most evident in individuals of low genetic quality (i.e., reflecting stable condition)^46,47^.

**Figure 1:**
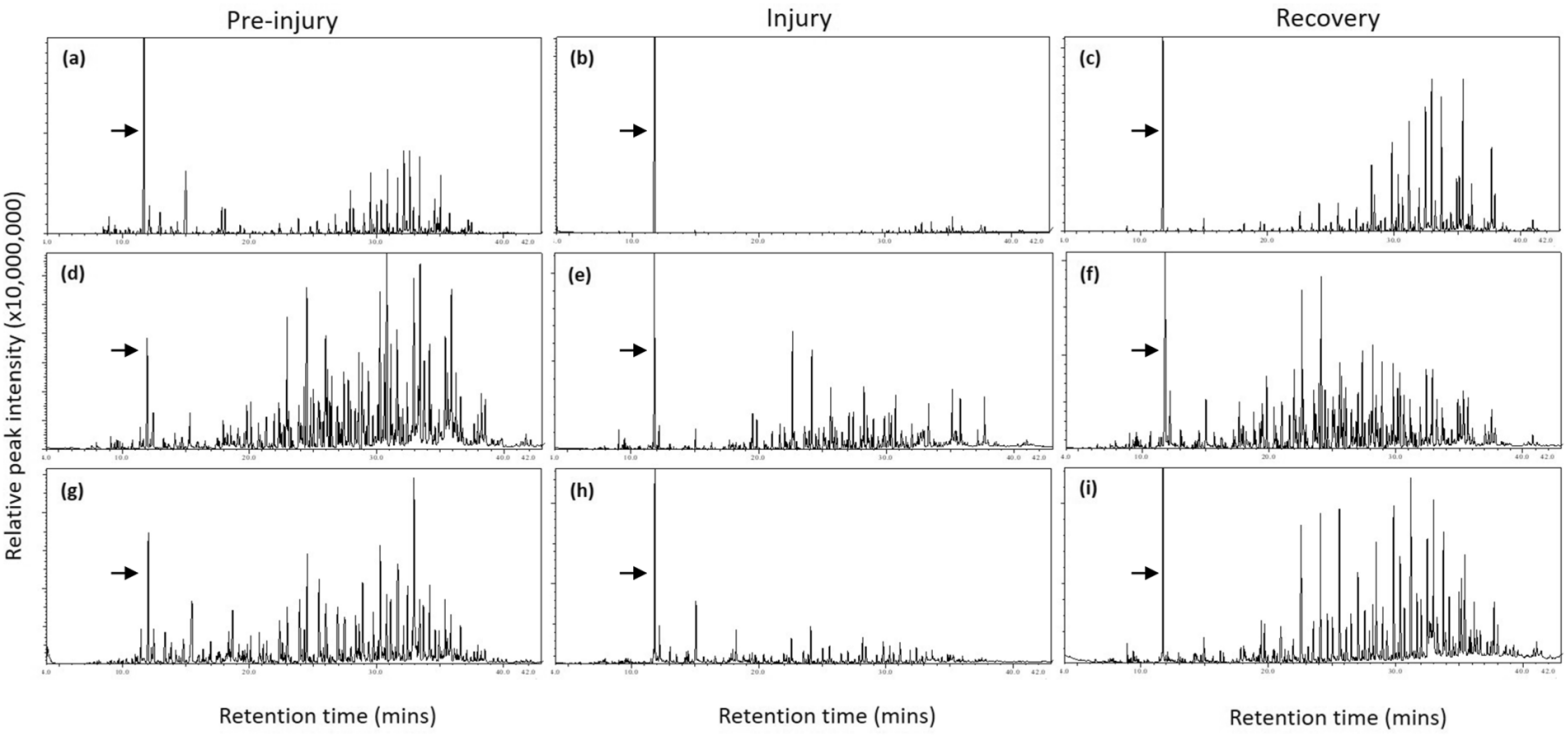
Representative gas chromatograms derived from the genital secretions of two male (**a**–**f**) and one female (**g**–**i**) ring-tailed lemur (*Lemur catta*), obtained during pre*-*injury (left), injury (center) and recovery (right) phases, showing that scent signatures are significantly depressed and altered during injury. All of these injuries were of ‘moderate’ severity: Sample (**b**) was collected one day post-injury, following a fight with group members resulting in hair pulls, a 1.5-cm laceration under the left eye, and a deep laceration to the right hand that required sutures. Sample (**e**) was collected two days post-injury from an animal with a fractured 4^th^ digit of the hind foot. Sample (**h**) was collected one day post-injury, following a fight with group members that resulted in puncture wounds to the right thigh and a 4.5-cm, shallow laceration to the right hand. Samples are scaled to the internal standard peak (hexachlorobenzene, *rt* 11.74 min; shown by arrows), except for (**d**) and (**g**), for which endogenous peaks were greater than that of the internal standard.

In addition, dominance and reproductive squabbles in lemurs are settled by the outcome of aggressive interactions^54^, such that intra- and inter-sexual aggression, resulting in naturally occuring injuries, can be relatively common. Along with increased scent-marking activity by both sexes, intra-male and intersexual aggression peak during the breeding season^55–59^; intra-female aggression also increases during the birthing and lactation seasons^56,60–63^. Physical aggression in ring-tailed lemurs is characterized by cuffs, lunges, chases and bites, occurring on the ground and in the trees^42,56,64^; subsequent injuries, whether from conspecifics or from falls, may be severe or even lethal^65^ (see Fig. 1b^60^). Injured animals, most often males, may pay both physical costs (e.g., physiological stress, injury) and social costs (e.g., loss of dominance status, reduced reproductive access, eviction from the group^60,62,65–68^). If olfactory signals are honest indicators of lemur condition that are costly to produce and maintain, then injured animals might be challenged to preserve normal scent signatures whilst their energetic resources are diverted towards recovery. Such costs in compromised animals could manifest as decreased chemical richness, decreased diversity and/or altered composition of odorants, relative to pre- or post-injury periods, and any of these chemical changes should be salient to conspecifics.

## Results

### Injuries in relation to season, the animal’s sex, and wound severity

We noted over twice as many nonlethal injuries in ring-tailed lemurs during the breeding season (*n* = 23) than during the nonbreeding season (*n* = 11), as well as slightly more injuries in males (*n* = 20) than in females (*n* = 14), but these differences were not statistically significant (all chi-squared tests *P* > 0.10). Nevertheless, these patterns are consistent with the significant seasonal and sex differences in injury reported in a previous study of the same population, but reflecting an earlier 35-year span from 1971–2006^60^. Injury severity, scored on a three-point scale (1 = ‘minor’, 2 = ‘moderate’; 3 = ‘severe’; see Materials and Methods), did not differ between seasons (mean severity scores, breeding season: 2.14; nonbreeding season: 2.07; Welch two sample *t*-test: *t*_34_ = 0.35, *P* = 0.73). In this female-dominant species, injuries sustained by females (mean severity score: 2.29) tended to be more severe than those sustained by males (mean score: 2.00; Welch two sample *t*-test: *t*_46_ = 1.46, *P* = 0.15). Both the time of year and the injured animal’s sex thus emerged as key variables affecting the likelihood of injury in lemurs.

### Consequences of injury on the chemical complexity of lemur scent signals

The chemical complexity of genital gland secretions was significantly altered during periods when lemurs were injured (‘injury’), relative to periods either before (‘pre-injury’) or afterwards (‘recovery’), when they were uninjured (Fig. 1; Table 1). Whilst controlling for potential covariates, such as injury severity or veterinary-prescribed antibiotic treatment in either sex, or hormonal contraception in females (see Materials and Methods), the specific patterns of chemical change by injury status varied by sex and season.

**Table 1:** Summary of the relationships between the chemical complexity of lemur genital gland secretions, as measured by the richness and Shannon diversity indices, and various explanatory variables by sex.

Sex	Explanatory variable	Richness			Shannon		
		*F*	df	*P*	*F*	df	*P*
Male	Injury status	*2.72*	*2, 36*	*0.078*	**3.98**	**2, 37**	**0.027**
	Season	0.76	1, 14	0.397	0.38	1, 15	0.546
	Severity	0.60	2, 37	0.552	1.66	2, 43	0.201
	ABX	0.99	1, 41	0.325	2.77	1, 41	0.012
	Injury status*season	**6.02**	**2, 35**	**0.006**	**5.06**	**2, 36**	**0.012**
Female	Injury status	1.57	2, 26	0.226	*2.91*	*2, 23*	*0.074*
	Season	2.97	1, 11	0.112	1.39	1, 10	0.264
	Severity	0.56	2, 11	0.560	0.56	2, 13	0.582
	ABX	0.75	1, 28	0.395	0.42	1, 25	0.525
	Contraception	**7.21**	**1, 9**	**0.024**	**5.77**	**1, 9**	**0.039**
	Injury status*season	0.99	2, 24	0.387	1.45	2, 21	0.267

The variables include the following: ‘injury status’ (pre-injury, injury, recovery); ‘season’ (breeding, nonbreeding); injury ‘severity’ (minor, moderate, severe); veterinary-prescribed ‘antibiotics’ (antibiotics, no antibiotics); and female hormonal ‘contraception’ (contracepted, not contracepted), as recommended by the Species Survival Plan. Significant results (*P* < 0.05) are indicated in bold type; trending relationships (0.05 < *P *< 0.08) are indicated in italics.

**Figure 2:**
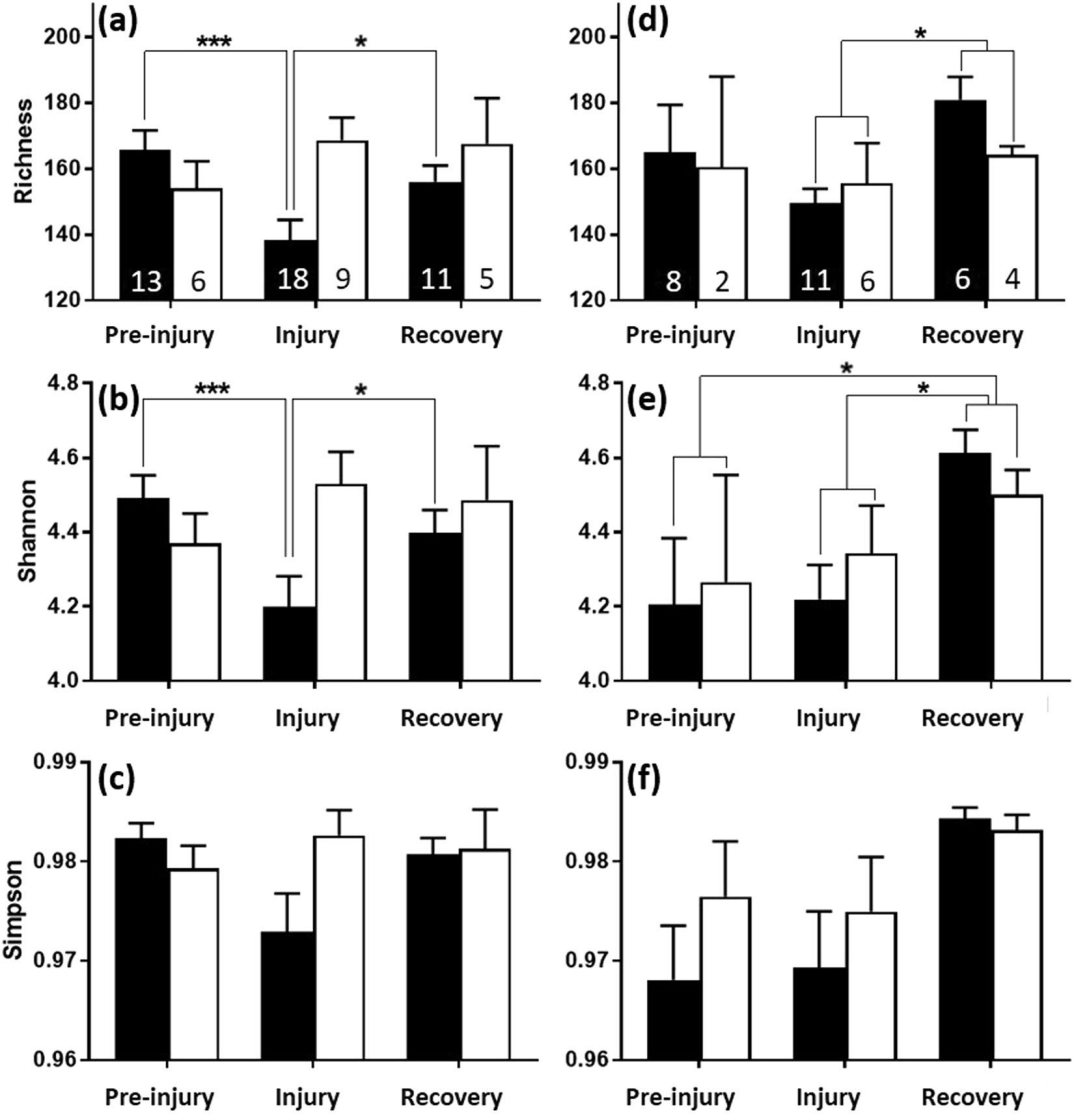
Mean + standard error chemical complexity of genital secretions collected from male (**a**–**c**) and female (**d**–**f**) ring-tailed lemurs during the breeding (black) and nonbreeding (white) seasons, across pre-injury, injury and recovery phases, showing significant effects of injury during the breeding season. Chemical complexity is measured using three diversity indices: Richness (**a**,**d**), Shannon (**b**,**e**) and Simpson (**c**,**f**). The numbers of samples are indicated in (**a**) and (**d**). Simpson data were too left-skewed to test robustly. Significant relationships are indicated as follows: ^*^P < 0.05; ^***^P < 0.001.

Notably, injuries occurring in males during the breeding season were associated with a significant decline in chemical complexity, as measured both by richness (pairwise contrasts, pre-injury vs. injury: *t*_36_ = 4.70, *P* < 0.001; injury vs. recovery: *t*_37_ = 2.93, *P* = 0.035; Fig. 2a) and by Shannon diversity (pairwise contrasts, pre-injury vs. injury: *t*_36_ = 5.92, *P* < 0.001; injury vs. recovery: *t*_37_ = 3.23, *P* = 0.014; Fig. 2b). The injury-induced changes in chemical richness represented, on average, a 10.6% loss in the number of compounds present. There were no significant differences in chemical richness or diversity between the males’ two ‘uninjured’ phases (Richness, Shannon indices, pre-injury vs. recovery: all pairwise contrasts *P* > 0.60). During the nonbreeding season, however, males showed no such injury-associated decreases in chemical complexity (Richness, Shannon indices, all pairwise contrasts *P* > 0.90). Although not directly tested because of data skewness, similar seasonally dependent declines in Simpson diversity during injury were also apparent (Fig. 2c). Regardless of season, the chemical complexity of male scrotal signals did not change significantly according to injury severity or concurrent veterinary-prescribed antibiotic treatment (Table 1; see Materials and Methods).

The chemical complexity of female labial signals also tended to vary with injury status (i.e., pre-injury, injury, recovery), but not exclusively with injury (Fig. 2d-f; Table 1). Although on average, chemical richness in females did not differ according to injury status, unexpectedly, *post-hoc* tests showed no significant difference in richness between samples collected prior to or during injury (pairwise contrasts, pre-injury vs. injury: *t*_25_ = 0.95, *P* = 0.61), but richness then increased significantly during recovery (pairwise contrasts, injury vs. recovery: *t*_26_ = 2.58, *P* = 0.02; Fig. 2d). Shannon diversity was also significantly greater during recovery, relative to pre-injury (pairwise contrasts, pre-injury vs. recovery: *t*_22_ = 2.73, *P* = 0.017) and injury (pairwise contrasts, injury vs. recovery: *t*_27_ = 2.86, *P* = 0.012; Fig. 2e). There was also no significant change in Shannon diversity during the period of injury, relative to pre-injury (pairwise contrast, pre-injury vs. injury: *t*_21_ = 0.079, *P* = 0.99). We observed similar patterns for the Simpson index (Fig. 2f). Both richness and Shannon diversity varied with female hormonal contraception, but not with concurrent antibiotic treatment, season or injury severity (Table 1).

### Consequences of injury on the chemical composition of lemur scent signals

**Figure 3:**
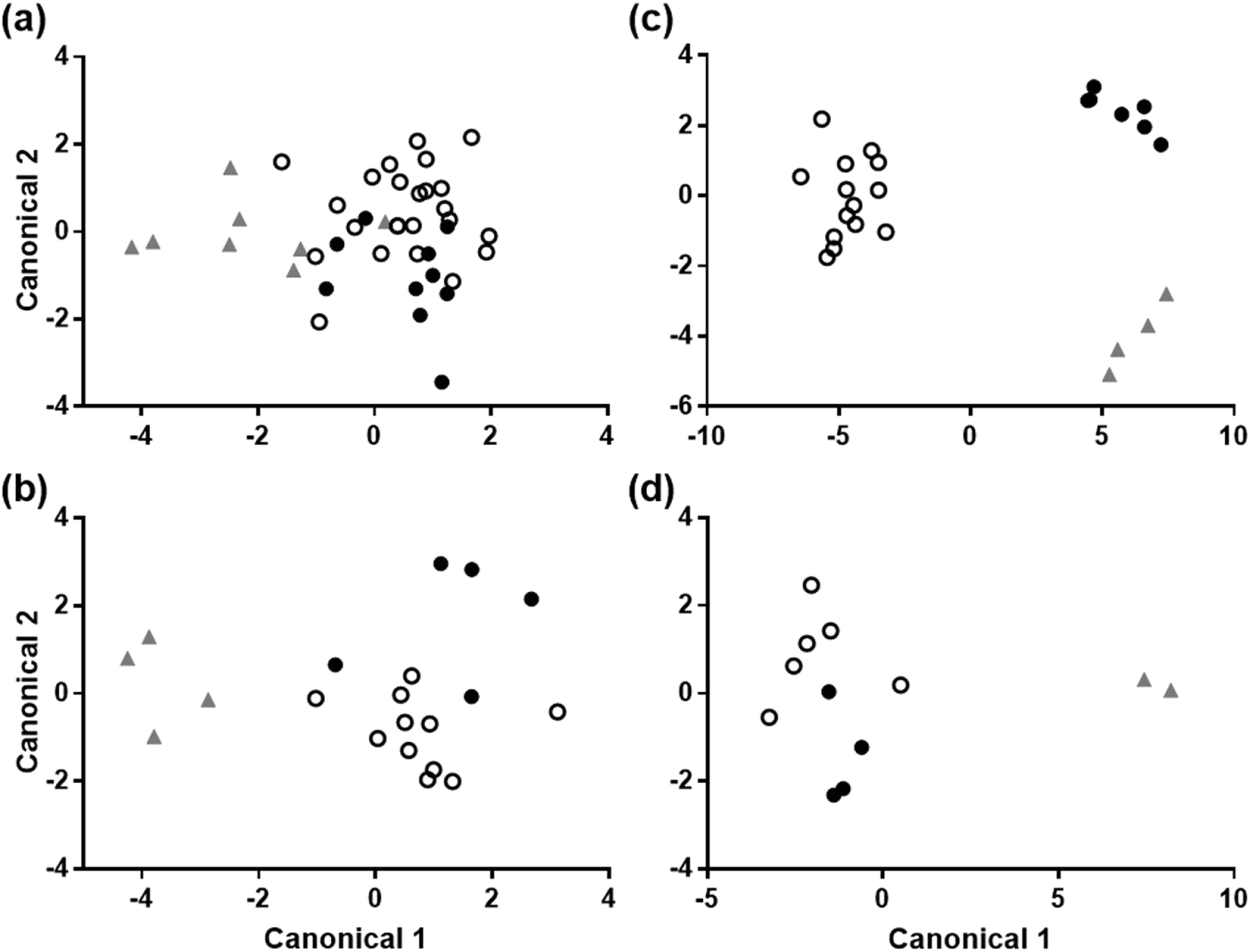
Representation of the differences between the chemical composition of genital gland secretions in ring-tailed lemurs that were uninjured (open circles), injured (filled circles) and injured receiving concurrent antibiotic treatment (shaded triangles). Shown are separate linear discriminate analyses for males in the breeding (**a**) and nonbreeding (**b**) seasons, and for females in the breeding (**c**) and nonbreeding (**d**) seasons.

The most common components of lemur genital secretions, as revealed by linear discriminate analyses (LDAs), varied according to whether the animals were uninjured, injured or injured and receiving concurrent antibiotic treatment. We retained principal components (PCs) with eigenvalues >1 separately for males and females, during the breeding and nonbreeding seasons (males, breeding season: *n* = 15 PCs, explaining 92.2% of variation across samples; males, nonbreeding season: *n* = 13 PCs, 97.6%; females, breeding season: *n* = 16 PCs, 95.8%; females, nonbreeding season: *n* = 10 PCs, 98.5%). In males, the LDAs for each subset of PCs correctly classified 73.8% and 90.0% of the samples collected during the breeding and nonbreeding seasons, respectively (breeding season: Wilks’ λ = 1.31, *P* = 0.19, Fig. 3a; nonbreeding season: Wilks’ λ = 0.94, *P* = 0.57; Fig. 3b). In females, the LDAs correctly classified 95.8% and 83.3% of samples collected during the breeding and nonbreeding seasons, respectively (breeding season: Wilks’ λ = 5.49, *P* < 0.001, Fig. 3c; nonbreeding season: Wilks’ λ not calculated due to small sample size, Fig. 3d).

Regarding overall chemical composition, male genital secretions varied with injury status in a season-specific manner (PERMANOVA main test, season*injury Pseudo-*F*_2,35_ = 2.17, *P* = 0.006). During the breeding season, the composition of scrotal secretions differed during injury compared with pre-injury (pairwise contrast, *t*_13_ = 1.59, *P* = 0.017); we did not find such a difference during the nonbreeding season (pairwise contrast, *t*_5_ = 1.25, *P* = 0.20). Scrotal secretions did not differ compositionally between injury and recovery phases, in either season (pairwise contrasts, all *P*s > 0.30). Overall chemical composition varied significantly depending on the individual animal (Pseudo-*F*_18,35_ = 2.14, *P* < 0.001), but not with antibiotic treatment (Pseudo-*F*_1,35_ = 0.78, *P* = 0.63) or injury severity (Pseudo-*F*_2,35_ = 0.53, *P* = 0.93). Random forests^69,70^, a type of classification tree analysis that assigns samples to categories (in this study, injury status) based on predictor variables (chemical compounds), did not reliably predict injury status in males.

In females, the overall composition of genital secretions tended to vary with injury status, although not significantly (PERMANOVA main test, injury status Pseudo-*F*_2,17_ = 1.43, *P* = 0.081), and this effect was not dependent upon season (PERMANOVA main test, injury status*season Pseudo-*F*_2,17_ = 1.05, *P* = 0.040). Overall composition varied depending upon the individual animal (Pseudo-*F*_10,17_ = 1.75, *P* < 0.001), but not with antibiotic treatment (Pseudo-*F*_1,17_ = 0.89, *P* = 0.54) nor injury severity (Pseudo-*F*_2,17_ = 0.43, *P* = 0.95). Lastly, random forests, based on overall labial secretion composition, correctly classified 71% of samples from females according to injury status. The three compounds contributing most to classification accuracy were fatty acid esters (*rt* 36.96 min, mol. wt. 508; *rt* 37.06 min, mol. wt. 452; *rt* 41.22 min, mol. wt. unknown): The first decreased during injury (mean relative abundance when uninjured: 0.24%; mean relative abundance when injured: 0.14%), the second was undetected in samples from injured animals and the third tended to increase during injury.

### Behavioural evidence of injury detection

**Figure 4:**
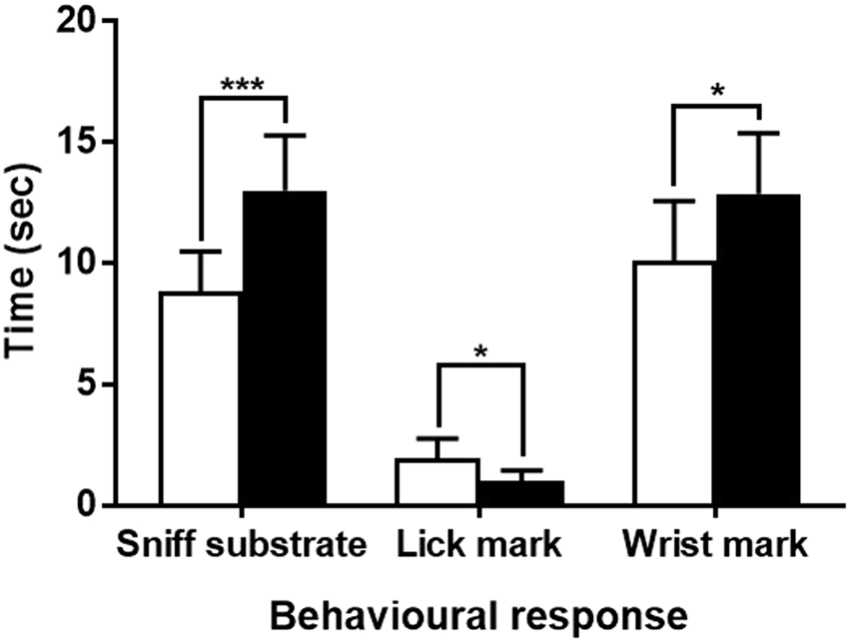
Mean + standard error behavioural responses showing discrimination by male ring-tailed lemurs between matched odorants from male conspecifics in either ‘uninjured’ (white) or ‘injured’ (black) condition. Shown are data from *n* = 15 bioassays. Significant relationships are indicated as follows: ^*^P < 0.05; ^***^P < 0.001.

Male ring-tailed lemurs (hereafter, the signal ‘recipients’) varied their investigation of and response to conspecific scent depending upon the injury status and sex of the animal from which the odorants derived (hereafter, the signal ‘donors’). When presented with two secretions from the same male donor, collected whilst the donor was ‘injured’ versus ‘uninjured’, the male recipients directed increased sniffing (*z* = 3.46, *P* < 0.001), decreased licking (*z* = 0.354, *P* = 0.043) and increased wrist-marking (*z* = 2.26, *P* = 0.024) to the scent of injured donors (Fig. 4). Whereas sniffing and licking are investigatory (potentially in response to volatile and nonvolatile components, respectively), wrist marking is a competitive form of counter marking^71^. We did not detect significant variation in other behavioural responses, nor did we observe any statistically significant differences in the behaviour of male recipients responding to the odorants from injured or uninjured female donors (all *P*s > 0.10). Neither the time the odorant had been in storage nor the number of trials in which the recipient had participated showed any relation to behavioural responses (all *P*s > 0.10).

## Discussion

Following long-term study of an aggressively female-dominant, group-living species – the ring-tailed lemur – we used an integrated analytical approach, to provide the first direct evidence of socially relevant changes in olfactory signals consequent to naturally occurring injury. When injured, ring-tailed lemurs of both sexes produced genital scent-gland secretions that were less complex than normal and altered in their chemical composition. Consistent with the proposition of energetic trade-offs affecting mechanistic pathways associated with signal production, these chemical deficits imply a cost to odorant production. Moreover, male signal recipients, relying on these odorants alone, could discriminate conspecific injury status and modulated their competitive behaviour accordingly, showing that honest olfactory advertisement of condition can have consequences on social behaviour.

Independent of any seasonal variations in injury severity, injury-induced alteration of male olfactory secretions was strongly evident during the breeding season and appeared to be associated with delayed recovery of the full suite of a signaller’s odours. By contrast, injury had only a weak, non-significant effect on a subset of male odorant components during the nonbreeding season. This differential effect may owe to seasonal patterns in the experience of physiological stress: Relative to the nonbreeding season, males in reproductive condition have raised concentrations of testosterone^56,57,59^ and corticosterone^59,72^, and show both increased scent-marking activity^55^ and heightened aggression^56,57,60,64^. During this intensely competitive time, male ring-tailed lemurs may be energetically challenged^46,56,72^ and thus less able to sustain the production of complex olfactory ornaments. Previously, we had observed males of low neutral heterozygosity being unable to sustain their normal signals during the breeding season^46^. Here, because our males were of average heterozygosity (see Supplementary Material), we instead suggest that injured males in reproductive condition had impaired ability to mitigate the physical costs of injury without drastically depleting their olfactory signatures.

In behavioural bioassays, male ring-tailed lemurs modulated their responses to the scents of male conspecifics depending upon their injury status, implying a function for condition assessment in same-sex competition. Examples of olfactory-based male assessment of competitor condition and fighting capacity are also found in lizards^73,74^, hamsters^75^ and laboratory mice^76^. When coupled with mechanisms for individual recognition^45,52^, sensitivity to changes in competitor condition helps avoid risks and fitness costs of unnecessary fighting by allowing males to (1) reliably assess competitor fighting ability, (2) assess their likelihood of winning an aggressive encounter with a potential competitor, and (3) selectively engage in aggressive interactions with animals of compromised or poor competitive ability^5,77,78^. Wrist marking and tail anointing are multimodal (i.e., combined visual, olfactory and, sometimes, auditory) displays of dominance in male ring-tailed lemurs^55,79,80^, whose dominance hierarchies are fluid. Status maintenance may thus require continual scent marking and assessment of competitor marks^46,80^. The higher rates of wrist marking we observed directed at odorants from injured animals (relative to odorants obtained when the same animals were healthy) is consistent with recipient males using counter-marking strategies to gain social dominance over competitively weak conspecifics^76,81^. Similarly, dominant resident males are more likely to engage in conspicuous tail-anointing and ‘stink-fighting’ behaviour, which functions as a potentially costly ‘badge of status’ relevant to both male and female recipients^79^. Alternately, injured animals might modulate their overt displays of aggression, including scent-marking behaviour, to avoid being attacked by dominant or otherwise healthy individuals^75^.

Unlike the situation in males, the genital secretions of female ring-tailed lemurs tend to increase in complexity from the nonbreeding season to the breeding season^43,47,82^, potentially suggesting some immunity of female signals to breeding season stressors. Nevertheless, as in males, females when injured produced genital secretions that differed chemically, albeit weakly, from those produced when they were uninjured, particularly during the breeding season. Although labial secretions also tended to be less chemically rich (but similarly diverse) during injury, lack of statistical significance could be attributed to a combination of both (1) the smaller number of samples available for females than for males and (2) considerable inter-individual variation in chemical diversity among females prior to injury. Alternatively, female olfactory signals may be differentially affected by injury: Females could have shown minimal decrements in chemical richness and complexity, but nevertheless experienced significant changes in the specific ratios of different compounds, such as the proportions of fatty acid esters. The latter have been shown to predict genetic quality^47^ and, now, injury status. Intriguingly, the complexity of female scent signatures tended to be greater during recovery, regardless of season, suggesting that, following an injury, females may express more chemically complex odorants than normal, perhaps to signal their return to vitality and to re-establish their dominance status within the group.

For female-dominant species, such as ring-tailed lemurs, signals for health and vitality that are sensitive to variations in physical condition could be of critical importance for female reproductive fitness^83,84^, analogous to condition-dependent signalling in males^83^. There are several qualitative and mechanistic similarities between male and female intra-sexual competition, that are intensified by group-living^85,86^. Female ring-tailed lemurs use scent marking in resource defence^87^ and to assert dominance over same-sex competitors^52,88^, whilst also closely monitoring the odours of other females^52^. Given that, as in males, female lemurs produce recognizable scent signatures^43^, discrimination of injury status and competitive ability could minimise the number of potentially costly, aggressive interactions undertaken with vigorous, healthy individuals^77,83^. Such recognition might therefore entail fitness benefits, both for signal producers and recipients. The functions and fitness consequences of olfactory signals in female competition are poorly understood, and may be improved with empirical studies across a range of taxa^85^. Endler^89^ notes that we often lack critical information, both about the relative importance of signals to different recipients and about the type of information being advertised. We suggest that this gap in our understanding is particularly true with regard to female signals. We would predict that female lemurs might be especially attentive to changes in the condition of other females during late gestation and lactation, when female competition and the fitness costs of losing aggressive encounters intensifies^49,56,60–63^. Evidence of female assessment of injury status in same-sex competitors might be found in other female-dominant species, including meerkats (*Suricata suricatta*) and spotted hyaenas (*Crocuta crocuta*), or in species that compete aggressively for resources and mates^83,84^.

A major challenge for the study of condition-dependent signals is to ensure that the type and degree of experimental stressor is biologically or environmentally relevant to the system in question^11,35^. In our study, the signaller’s condition was naturally altered during periods of injury, and the physiological and energetic trade-offs associated with injury were amplified during the breeding season. The production of condition-dependent chemical signals may share metabolic pathways with critical cellular processes^12^, such as mitochondrial respiration^14,90^. Signal production pathways might also become limited by perturbations in insulin-like growth factors^23,91^, or by oxidative stress^4,92^, immune activation^10,31,32^ or inflammation^93^. For example, injury induces the production of protein complexes, termed ‘inflammasomes’, that are involved in the inflammatory response and tissue repair, and trigger pyroptosis, a form of cellular death^94^. Such processes could contribute to an ‘injury-specific’ odour, akin to disease-specific changes in body odour described in human patients^95^. Data on the concurrent physiological state of signallers are needed to describe the specific mechanisms underlying honest, semiochemical production.

Although researchers have previously argued that cost is not necessary to maintain honesty^7,12,15^, we suggest that our study provides strong evidence of trade-offs in resource allocation during injury. Moreover, a cost to lemur odorant production could function as a ‘revealing handicap.’ Changes in the production of potentially costly compounds, such as lipids and fatty acid esters, may be mediated by energetic trade-offs between immunological and physiological regulation of somatic repair following injury, and allocation of essential nutrients to chemical signal production^46,47^. Additionally, the genetic quality of individuals will likely affect both their condition and ability to buffer the effects of environmental stressors^11,13^. We echo previous calls for more empirical research in a variety of taxa and signalling systems^23^, with such research incorporating both observational and manipulative approaches^18^ to better tease apart the non-mutually exclusive influences of genotype, costs and condition-dependent signals.

Lastly, along with becoming altered during injury, lemur odorants were further perturbed by concurrent veterinary-prescribed antibiotic treatment, consistent with the putative removal of fermentative bacteria crucial for odour production^96^. In both males and females, antibiotic treatment was associated with concurrent changes in the composition of the most commonly occurring volatile chemical components, but not with changes in overall chemical complexity or composition. These results suggest that the most widespread compounds in lemur genital secretions may be modified and/or produced, at least in part, by bacteria and, consequently, are sensitive to broad-spectrum antimicrobials. Because the composition of commensal microbes shaping an individual’s scent signature might be altered by infection or host health^6^, elucidating the specific contribution of bacteria to lemur social odours requires an experimental approach in healthy animals.

By using a unique system involving natural alteration of the physical condition of the signalling animal, our study provides, to our knowledge, the first supporting evidence for a socially relevant olfactory indicator of naturally occurring injury. Given the social and physical costs of injury, particularly in an aggressively female-dominant species, lemurs of both sexes could benefit from being attentive to the health status of conspecifics and being selective about engaging in aggressive behaviour with specific individuals. Further research will elucidate the specific mechanisms by which physical injury can alter sexual signals, including potential and realised costs associated with the production of condition-dependent signals.

## Materials and Methods

### Subjects and housing

Our subjects were 27 adult ring-tailed lemurs (17 males, 10 females; mean ± standard error or s.e. age at the time of study: 7.61 ± 0.53 yrs, range: 1.8–25.5 yrs). Of these, 23 (13 males and 10 females) provided genital secretion samples, collected between 2007–2016 (Table 2), and nine males served as focal subjects in behavioural bioassays, conducted in 2016 (see sections on sample collection and behavioural bioassays, below). All of the subjects were captive-born and housed socially at the Duke Lemur Center (DLC; Durham, NC, USA)^43,56^. The animals’ social housing conditions allow for exposure to conspecific visual, auditory and olfactory cues, as well as for naturally occurring interactions, including those of aggression and their subsequent injuries (see injury section, below)^48,56,60^. Most subjects are semi-free ranging, with access both to forested outdoor enclosures (3–7 ha) and to temperature-controlled, indoor areas. A minority of subjects are housed indoors year-round. All of the animals are provided with a mixed diet of commercial primate chow, fruit, vegetables, fresh browse, and water^56,60^. Our research protocols (Protocol Registry Numbers A232–06–07, A171-09-06, A143-12-05 and A111-16-05) abided by the regulations of the United States Department of Agriculture and were approved by the Institutional Animal Care and Use Committee of Duke University. The DLC is fully accredited by the American Association for the Accreditation of Laboratory Animal Care; information on the DLC’s conservation, education and research mission is available at http://lemur.duke.edu/.

**Table 2:** Number of unique injury events, involving three phases, that are represented by odorant samples collected from adult ring-tailed lemurs at the Duke Lemur Center, from 2007–2016.

Sex	Season	Phases (sampled for GC-MS, and bioassays)		
		Pre-injury	Injury	Recovery
Male	Breeding	13 (13, 10)	14 (18, 15)	11 (11, 5)
	Nonbreeding	6 (6, 0)	6 (9, 1)	5 (5, 1)
Female	Breeding	8 (8, 3)	9 (11, 3)	6 (6, 0)
	Nonbreeding	2 (2, 7)	5 (6, 11)	4 (4, 4)
Total		29 (29, 20)	34 (44, 30)	26 (26, 10)

Shown in parentheses are the numbers of samples used for gas chromatography-mass spectrometry (GC-MS) and behavioural bioassays, respectively. These samples were obtained at one time point during each uninjured phase (pre-injury and recovery), but at one or more time points during each injury phase.

### Injury identification, occurrences, and classification

All DLC animals are monitored closely each day: If an aggressive interaction is directly observed or suspected, the individuals most likely to be involved are captured for closer examination and, if necessary, veterinary treatment. In the case of severe wounding or continued targeted aggression, an animal may be temporarily or permanently removed from its group. Veterinary records are added to a medical records database (Species360, Bloomington MN), detailing the nature and severity of any injuries, their cause (if known), any prescribed medications, follow-up care and treatment outcomes. We communicated with the veterinary staff about all injuries, as they presented, but retrospectively used the Species360 database to verify the condition for all of our subjects.

We report on 34 unique injury events (affecting 13 male individuals and 10 females, with some animals being injured multiple times during the study; Table 2). Most (28/34 or 82%) resulted from fights or probable fights with members of the animal’s own group, but altercations also occurred between members of neighbouring groups. Of the injuries resulting from fights, those sustained by males were most often inflicted by other males (*n* = 10 or 62.5%), but also by females (*n* = 2) or an unknown assailant (*n = *4). The remaining injuries in males (*n* = 4) owed to unknown causes. Injuries in females were inflicted or likely inflicted by other females (*n* = 7) or unknown assailants (*n* = 5), or resulted from accidents (*n* = 2).

Some injuries (*n* = 5) changed in severity over the course of treatment (i.e., they improved as infections cleared or worsened with subsequent amputations or infections). We thus differentiated 39 injuries based on a three-point scale of severity: ‘Minor’ injuries (*n* = 8) included superficial scrapes, hair pulls, punctures or small (< 2 cm) lacerations requiring minimal veterinary intervention; ‘moderate’ injuries (*n* = 21) included lacerations (2–10 cm), digit fractures or dislocations; ‘severe’ injuries (*n* = 10) included fractures, amputations, and lacerations (> 2 cm) that damaged tendons or other deep tissues. Some injuries (minor: *n* = 2; moderate: *n* = 7; severe: *n* = 8) subsequently became infected and required antimicrobial treatment (most commonly involving amoxicillin or enrofloxacin, both of which are broad-spectrum antibiotics).

Individuals were uninjured during the periods before and after each injury (‘pre-injury’ and ‘recovery’ phases, respectively). The latter occurred minimally two weeks after the initial insult, as determined from veterinary records indicating that the subject was in good health (i.e., the wound had healed, there was no new evidence of trauma, no pathological signs of disease or illness, and normal behaviour had resumed).

### Odorant sample collection

All odorant sampling of lemur genital (i.e., male scrotal and female labial) secretions occurred in triplicate (i.e., three swabs were taken), following previously published procedures^43^ (see Supplementary Material). We later divided the replicate samples for use in GC-MS analyses and in behavioural bioassays (Table 2; for details on the analytical and bioassay procedures, see below). In early years of the study, we routinely (i.e., monthly) collected odorants from all healthy ring-tailed lemurs at the DLC throughout the breeding (November–February in the northern hemisphere^56^) or nonbreeding seasons (March–October). These samples provided the pool from which we selected all of the ‘pre-injury’ and many of the ‘recovery’ samples. Additionally, we opportunistically collected ‘injury’ odorants when an animal was brought in for veterinary care, typically on the day the insult occurred or shortly afterwards, whilst the animal was still showing active signs of injury (mean ± s.e.: 6.25 ± 0.90 days post-insult, range: 0–26 days). In later years of the study, sample collection was more sporadic, specifically targeting the collection of injury or recovery samples. The mean (± s.e.) time span between collection of pre-injury vs. injury samples was 1.36 ± 0.18 years (range: 10 days – 6 years) and between collection of injury vs. recovery samples was 1.42 ± 0.27 years (range: 14 days – 6 years). The maximum time span across all sampling relating to a single injury was eight years.

Obtaining complete sequences of samples for a given injury (representing pre-injury, injury, and recovery) was not always feasible owing to logistical challenges, including matching the samples by season or female reproductive state (see section on statistical analyses, below). We obtained 21 complete sequences; nevertheless, for each injury, we minimally obtained one set of samples during an uninjured phase, either before or after the injury. In 9 of the 34 unique injuries described, we also collected additional (*n* = 1–3) triplicate sets of odorant samples when the veterinary staff reassessed injury severity during follow-up examinations. Such resampling allowed us to track changes in chemical composition, either as the severity of the injury changed or as the animal began receiving antibiotics.

### Gas chromatography-mass spectrometry

We used our previously published GC-MS procedures^43,46^ (see Supplementary Material) to describe the chemical composition of lemur odorants collected during pre-injury, injury, and recovery phases (Table 2). Because it is not possible to control for the absolute amount of secretion collected or analysed, we present data on relative abundances. We have previously shown that individual-specific lemur scent signatures are stable across years and with storage time^48,52^, which we also verified here (see Supplementary Fig. S1).

### Behavioural bioassays

We performed 30 behavioural bioassays^52,71^ within a five-day period in late October 2016, at the onset of the breeding season for ring-tailed lemurs in the northern hemisphere^56^. We used male recipients because they respond reliably to scents from both males and females^52^ and, during the breeding season, respond most strongly^52^ and scent mark most intensively^55^. We presented nine healthy male ‘recipients’ (each receiving 1–5 bioassays) with a choice between two odorants collected from a given, conspecific ‘donor,’ one collected whilst the donor was uninjured and one whilst injured (Table 2; see Supplementary Material). Nine males and six females served as odorant donors (in 1–4 bioassays each). Five of our recipient males also acted as odorant donors. We presented recipients only with the odorants from unfamiliar donors (defined as never having belonged to the recipient’s group and whose odorants had not been encountered by the recipient in the previous two years). We controlled for seasonal variation in odorant composition by matching the paired odorants according to the timing of sample collection (i.e., where possible, we used samples collected within the same calendar months, even if collected across years). We also did not use odorants from individuals concurrently treated with antibiotics or from females that were either pregnant, lactating or hormonally contracepted.

### Statistical analyses

We first investigated the chemical complexity of odorants deriving from uninjured versus injured lemurs by calculating the following three indices for each odorant type: Richness, Shannon and Simpson^46,97^. Richness is simply the number of peaks retained for statistical analyses, whereas the Shannon and Simpson indices apply weight to peaks based on their relative abundance: The Shannon index is most sensitive to those peaks of intermediate abundance, whereas the Simpson index is most sensitive to the most abundant peaks^97^. Shannon and Simpson indices were calculated using the *vegan* package (version 2.4–4^98^) in the program R^99^ and R Studio (version 1.0.136^100^). For these analyses, we controlled for seasonal variation and individual-specificity in chemical profiles by matching ‘pre-injury,’ ‘injury,’ and ‘recovery’ odorants collected sequentially from the same animal within either the breeding or nonbreeding seasons. We further controlled for female reproductive status by (1) matching odorants according to female contraceptive treatment (contracepted, not contracepted) and (2) not using odorants from pregnant or lactating individuals. When possible, we matched odorants collected within the same season (*n* = 19 injuries); other injuries were seasonally matched with odorants collected across years (*n* = 15).

To test for variations in chemical complexity between injured and uninjured animals, we analysed each chemical diversity measure separately in a series of general linear mixed-effects models (GLMMs) in the *lme4* package in R (version 1.1–14^101^). Because males and females have different genital secretions (i.e., scrotal vs. labial^43^) and show different seasonal patterns in chemical complexity^46,82^, we analysed the data for each sex separately. Although group composition and size varied over the course of the study, this variance was not likely to impact our results, as we have previously found no significant effects of housing condition on lemur chemical profiles^48,82^. We therefore excluded aspects of group composition from the analyses. Similarly, we also excluded animal age and dominance rank, as previous studies in adult ring-tailed lemurs have not shown these variables to significantly affect odorant composition^43^ (see Supplementary Fig. S1), or their salience to conspecifics^52^. In each GLMM, we included as fixed effects the animal’s injury status (pre-injury, injury, recovery), season (breeding, nonbreeding), and their interaction, along with injury severity (minor, moderate, severe), and female contraceptive treatment (contracepted, not contracepted). Some animals were prescribed antibiotics as part of their veterinary treatment: Because the ‘fermentation hypothesis’ posits that beneficial microbes inhabiting animal scent glands contribute to host social odours^96,102^, we additionally controlled for concurrent antibiotic treatment (antibiotics, no antibiotics). A random effect, ‘identity’, was also included, which incorporated both the animal’s identity and its specific injury, thus controlling for cases of multiple injuries for some individuals (e.g., 19 injuries derive from eight individuals). The significance of all fixed effects was assessed using *t*-statistics and degrees of freedom (Satterthwaite approximation) estimated in the *lmerTest* package (version 2.0–33^103^) in R. To confirm the robustness of our models, we verified the normality of residuals using Q-Q plots and Shapiro-Wilk tests. We tested the significance of *post hoc* contrasts using Tukey-adjusted *P-*values (*multcomp* package version 1.4–8^104^). We used Gaussian distributions and applied transformations to Shannon index data for males and females (^2 and ^5 respectively, to improve skewness). Simpson data were too skewed to test robustly, but for comparative purposes we present the mean ± s.e. for all three indices.

To address the possibility that two scent samples could be equally complex, but different in composition, we next examined changes in lemur odorant composition associated with being uninjured, injured, and injured whilst receiving concurrent antibiotic treatment. As in our analyses of chemical diversity, we divided the chemical data on relative abundances into four subsets (one for each sex in each season) and conducted separate multivariate statistical analyses for each subset. First, using linear discriminate analysis (LDA), we tested for changes in composition based on the most common chemical components. To reduce the dimensionality of the data, we also calculated principal components (PCs), using the relative abundance of peaks occurring in minimally 80% of each data subset. We combined samples collected ‘pre-injury’ and during ‘recovery’ into a single ‘uninjured’ category, thereby reducing the number of classification categories. We used PCs with eigenvalues >1 as variables in LDAs, classifying samples according to the injury status of the donor animal (i.e., ‘uninjured,’ ‘injured’, ‘injured + ABX’). We calculated PCs and LDAs using JMP (version 13).

We next tested for differences in the overall composition of chromatograms (retaining peaks that comprised >0.05% of the total chromatogram area and occurring in >5 samples), using a permutational MANOVA (‘PERMANOVA’) in Primer-E (version 7.0.13^105^) with the PERMANOVA+ add-on^106^. We first applied a square root transformation to reduce the influence of the most abundant peaks, then calculated a Bray-Curtis dissimilarity matrix. PERMANOVAs were performed using type III sums of squares, a reduced fit model procedure and 9999 permutations. As in the analyses of odorant diversity, we included as fixed factors the interaction between animal injury status and season, plus antibiotic treatment, contraceptive treatment (females only) and injury severity, along with identity as a random factor.

Lastly, we used random forests^69^, each comprising 1000 classification trees, to identify the compounds most affected by being injured (versus uninjured) and, thus, potentially costly to produce. For these analyses, we again pooled the ‘pre-injury’ and ‘recovery’ phases to minimize the number of categories, and used the *randomForest* package^107^ in R. We generated random forests separately for males and females, and excluded from our analyses the samples obtained from animals treated with antibiotics. We report on compounds that contributed most to model classification accuracy (i.e., we minimized ‘out-of-bag’ error rate^70^). We compared model accuracy with that of a ‘default’ model, which simply assigns all samples to the most numerous class.

To test for differences in male behavioural responses to odorants derived from injured versus uninjured donors, we used GLMMs and the *glmmADMB* package (version 0.8.3.3^108^) in R. Due to the limited number of bioassays we could perform, we could not robustly test for differences in male responses depending upon both the sex and reproductive condition of the donor animal. Therefore, we restricted our analyses to bioassays involving odorants collected from females during the nonbreeding season (*n* = 11 bioassays) and from males during the breeding season (*n* = 15). We ran separate GLMMs for each of these two donor categories, using each behavioural response as the dependent variable. We scored behaviour (sniff, lick, wrist mark) as counts and/or as bouts of varying duration (seconds per response). Tail marking, biting and ‘threat yawns’ were not observed sufficiently often to analyse robustly. We used Poisson, negative binomial or gaussian distributions as appropriate. In each GLMM, we included the sample’s relative storage time (i.e., whether it was the ‘older’ or ‘newer’ of the pair) and trial number (i.e., the total cumulative number of bioassays in which the recipient had participated) as fixed factors, and donor identity nested within recipient as a random factor. We used a stepwise GLMM selection procedure, sequentially dropping variables with the greatest *P*-value from the GLMM, until only significant factors remained. We then added each excluded factor back into the final model to confirm statistical non-significance^109^. In all of these statistical analyses, we set significance at α < 0.05.

### Data accessibility

GC-MS odorant sample data and bioassay. ‘CSV’ files are available in the *Dryad* repository, at 10.5061/dryad.9b37gv4.

## Electronic supplementary material

Supplementary Information
