# Supplementary material for: Costs of injury for scent signalling in a strepsirrhine primate
Source: Sci Rep. 2018 Jun 29;8:9882. doi: 10.1038/s41598-018-27322-3 (PMC6026195; doi:10.1038/s41598-018-27322-3)
Supplement: Supplementary file 1 — Supplementary Information [file 41598_2018_27322_MOESM1_ESM.pdf]

Costs of injury for scent signalling in a strepsirrhine primate

Supplementary Material

Rachel L. Harris[1], Marylène Boulet[2], Kathleen E. Grogan[3], Christine M. Drea*[1,4]

[1]Department of Evolutionary Anthropology, Duke University, Durham, NC, USA
[2]Department of Biology, Bishop's University, Sherbrooke, QC, Canada
[3]Department of Anthropology, Pennsylvania State University, State College, PA, USA
[4]Department of Biology, Duke University, Durham, NC, USA

Supplementary Methods

Odorant sample collection and analysis. We collected odorants following established procedures whilst the animal was gently restrained by trained Duke Lemur Center (DLC) staff or anaesthetized during veterinary examination[1]. Briefly, using pre-washed cotton held with sterile forceps, we swabbed the glandular field of either the scrotum or labia of our subjects. The samples were immediately stored in individual pre-washed chromatography vials, placed on ice whilst at the DLC (up to 2h), then transferred to -80 °C until the sample was either analysed via gas chromatography-mass spectrometry (GC-MS) or used in behavioural bioassays. We collected 'control' odorants by exposing a clean cotton swab to air before storing it on ice in a pre-washed chromatography vial.

We extracted odorant samples in methyl *tert*-butyl ether (MTBE) and concentrated them under compressed nitrogen flow to a final volume of 50-80 µL. All of the samples were analysed using a GC-MS-QP-2010 instrument (Shimadzu Scientific Instruments) equipped

with a Shimadzu AOC-20i auto-sampler and an Agilent DB-5MSUI (30 m × 0.25 mm × 0.25 µm) column. To improve peak separation and resolution, we modified our existing protocol[1,2] by increasing the scan speed to 5000 amu/sec and the scan range to 50-550 *m/z* (total run time: 43 min). We also calculated retention indices by running a saturated alkanes mixture (49452-U, Sigma-Aldrich) under the same analytical conditions. We analysed a ‘control’ sample after every six odorant extracts to check for carry-over contamination between samples. To standardize retention times (*rt*) between samples, we aligned the peaks of two standards: 1 mg/mL hexachlorobenzene (HCB, *rt* 11.74 min, 5 µL added to the concentrated sample prior to analysis), and squalene (*rt* 28.22 min), an endogenous product commonly found in lemur secretions[2].

Because we modified our GC-MS temperature protocol and used a new GC column to improve peak separation, we observed consequent shifts in degrees of overlap among co-eluting peaks and relative retention times compared with previous studies[1,2]. For example, we detected more compounds in males (*n* = 298 peaks), but fewer compounds in females (*n* = 272), than previously reported[1.] These differences likely result from our increased ability to detect peaks of low abundance, which may not have reached previous quantitation thresholds, but we detected fewer alcohols, a known component of female genital secretions[3].

Although we have previously verified that time spent in cold storage does not appreciably affect chemical diversity or composition, we have not previously compared samples collected over a period of more than 2-3 years[4]. To confirm (1) sample preservation over storage time (regardless of sample state, i.e., as a cotton swab or as an extract), and (2) temporal stability of lemur odorant composition, we compared chromatograms derived from duplicate samples collected from an adult male and an adult female lemur in 2007-2008 and 2014, then analysed in 2016. Gas chromatograms presented in earlier studies were generated using a Restek GC column[2,5], whereas the 2016 GC-MS analyses were completed using the

same GC-MS instrument, but with an Agilent column. Because the number of peaks detected varied according to the temperature protocol and GC column, we consistently measured chromatogram peak sizes according to our original protocols[1,2]. All chromatograms were broadly similar in appearance, richness and composition, and we did not observe clear differences in the relative peak abundances (Fig. S1).

Behavioural bioassays. For each bioassay, we temporarily isolated the recipient animal from its group members. We then rubbed two thawed genital odorant samples onto a 1 cm^2 section of one of two dowels, to simulate a naturally placed scent mark, and simultaneously inserted both dowels into the enclosure, placed approximately 50 cm apart. We then secured the dowels to the enclosure fencing at a 60° angle to the ground. We filmed each bioassay (HDRCX405, Sony video camera), beginning prior to dowel placement. Trials lasted 10 minutes from the placement of the dowels in the enclosure. Recipients were not presented with odorants from the same donor more than once and did not participate in more than three bioassays per day. We randomized the placement (left or right) of the 'uninjured' and 'injured' samples and the order of odorant presentations. Each odorant sample was used only once.

Using a published ethogram of distinctive, mutually exclusive behaviour[6], a scorer blind to the scent treatments recorded recipient behaviour from the video. The behaviour scored included sniffing, licking, marking, and time spent in proximity to the odorants. Investigatory behaviour, such as sniffing or licking directed at either the 'scent mark' or the entire dowel, may indicate interest or effort required to interpret the information present in an odorant, whereas marking (e.g., wrist marking, genital marking, tail marking) indicates a specific response to the odorant[7,8]. We assessed intra-observer reliability by calculating an index of concordance[9]: once overall concordance exceeded 95% during training, between the

scores from the observer and an experienced trainer, all videos were scored in a random order. We also assessed intra-observer reliability across the video scoring period, by scoring three videos twice: The indexes of concordance for each behaviour scored across the two datasets were as follows: sniff substrate 92%; lick substrate 96%; lick mark 100%; sniff mark 86%; wrist mark 100%; shoulder rub 80%; time in general proximity 96%.

Study subject neutral heterozygosity. Of our 15 males and 10 females, we obtained H_o values for 8 and 7 of them, respectively. Their means (males: 0.51 ± 0.05; females: 0.53 ± 0.04) and ranges (males: 0.21-0.64; females: 0.36-0.64) are consistent with the values previously obtained for a larger population[2,3]. We therefore would not describe the animals in our study as being of particularly low genetic quality.

Supplementary References

1. Scordato, E. S., Dubay, G. & Drea, C. M. Chemical composition of scent marks in the ringtailed lemur (*Lemur catta*): glandular differences, seasonal variation, and individual signatures. *Chem. Senses* **32**, 493-504 (2007).
2. Charpentier, M. J. E., Boulet, M. & Drea, C. M. Smelling right: the scent of male lemurs advertises genetic quality and relatedness. *Mol. Ecol.* **17**, 3225-3233 (2008).
3. Boulet, M., Crawford, J. C., Charpentier, M. J. E. & Drea, C. M. Honest olfactory ornamentation in a female-dominant primate. *J. Evol. Biol.* **23**, 1558-1563 (2010).
4. Crawford, J. C., Boulet, M. & Drea, C. M. Smelling wrong: hormonal contraception in lemurs alters critical female odour cues. *Proc. R. Soc. B* **278**, 122-130 (2011).
5. Boulet, M., Charpentier, M. J. & Drea, C. M. Decoding an olfactory mechanism of kin recognition and inbreeding avoidance in a primate. *BMC Evol. Biol.* **9**, 281 (2009).
6. Grogan, K. E., Harris, R. L., Boulet, M. & Drea, C. M. Genetic variation at the MHC influences both olfactory signals and scent discrimination in ring-tailed lemurs. *bioRxiv* doi: 10.1101/337105 (2018).
7. Drea, C. M. D'scent of man: A comparative survey of primate chemosignaling in relation to sex. *Horm. Behav.* **68**, 117-133 (2015).

8. Greene, L. K. *et al.* Mix it and fix it: functions of composite olfactory signals in ring-tailed lemurs. *Roy. Soc. Open Sci.* **3**, 160076 (2016).
9. Martin, P. & Bateson, P. Measuring behaviour (Cambridge University Press, Cambridge, UK, 1993).

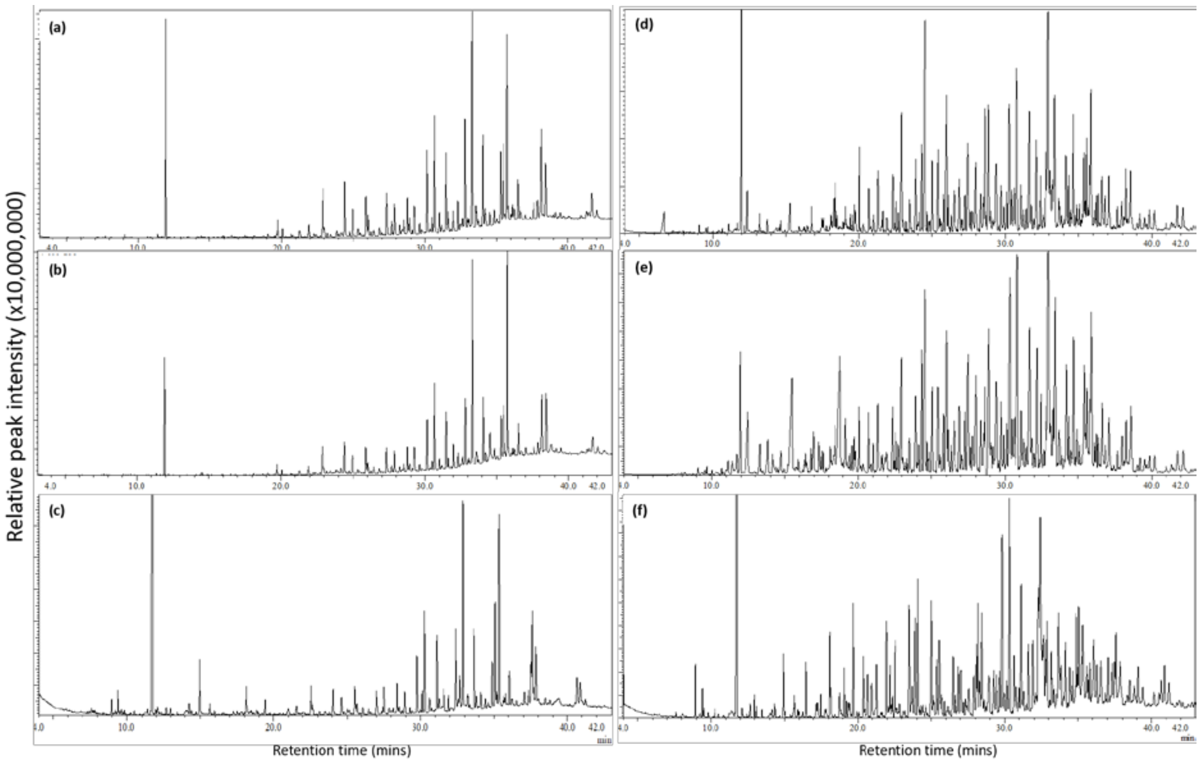

Figure S1. **Representative gas chromatograms of genital secretions, that were collected from a male (left) and female (right) ring-tailed lemur, showing chemical stability across storage periods despite samples being stored and analysed at different times.** The samples represented in (**a,d**) were collected in 2007 and 2008 respectively, then extracted and analysed by gas chromatography-mass spectrometry (GC-MS) in 2016; (**b,e**) were collected and extracted in 2007 and 2008, then analysed by GC-MS in 2016; (**c,f**) were collected and extracted in 2014, then analysed by GC-MS in 2016.
